# The Retinoic Acid Receptor Beta (*Rarb*) Region of *Mmu14* Is Associated with Prion Disease Incubation Time in Mouse

**DOI:** 10.1371/journal.pone.0015019

**Published:** 2010-12-06

**Authors:** Julia Grizenkova, Shaheen Akhtar, John Collinge, Sarah E. Lloyd

**Affiliations:** MRC Prion Unit and Department of Neurodegenerative Diseases, UCL Institute of Neurology, University College, London, United Kingdom; Johns Hopkins, United States of America

## Abstract

In neurodegenerative conditions such as Alzheimer's and prion disease it has been shown that host genetic background can have a significant effect on susceptibility. Indeed, human genome-wide association studies (GWAS) have implicated several candidate genes. Understanding such genetic susceptibility is relevant to risks of developing variant CJD (vCJD) in populations exposed to bovine spongiform encephalopathy (BSE) and understanding mechanisms of neurodegeneration. In mice, aspects of prion disease susceptibility can be modelled by examining the incubation period following experimental inoculation. Quantitative trait linkage studies have already identified multiple candidate genes; however, it is also possible to take an individual candidate gene approach. *Rarb* and *Stmn2* were selected as candidates based on the known association with vCJD. Because of the increasing overlap described between prion and Alzheimer's diseases we also chose *Clu*, *Picalm* and *Cr1*, which were identified as part of Alzheimer's disease GWAS. Clusterin (*Clu*) was considered to be of particular interest as it has already been implicated in prion disease. Approximately 1,000 heterogeneous stock (HS) mice were inoculated intra-cerebrally with Chandler/RML prions and incubation times were recorded. Candidate genes were evaluated by sequencing the whole transcript including exon-intron boundaries and potential promoters in the parental lines of the HS mice. Representative SNPs were genotyped in the HS mice. No SNPs were identified in *Cr1* and no statistical association with incubation time was seen for *Clu* (P = 0.96) and *Picalm* (P = 0.91). Significant associations were seen for both *Stmn2* (P = 0.04) and *Rarb* (P = 0.0005), however, this was only highly significant for *Rarb*. This data provides significant further support for a role for the *Rarb* region of *Mmu14* and *Stmn2* in prion disease.

## Introduction

Prion diseases, also known as transmissible spongiform encephalopathies, are fatal neurodegenerative disorders of both humans and animals. They include Creutzfeldt-Jakob disease (CJD) in humans, bovine spongiform encephalopathy (BSE) in cattle and scrapie in sheep [1]. They are characterized by prolonged incubation periods and distinctive neuropathology that includes deposition of an abnormal form of the prion protein (PrP^Sc^), spongiform change, gliosis and neuronal loss.

Human prion diseases may be described as inherited, sporadic or acquired. Inherited prion disease accounts for ∼15% of human prion disease and is caused by mutations in the prion gene (*PRNP*). Sporadic CJD accounts for ∼85% of human prion diseases and has no known cause. Prion diseases are also transmissible (acquired) and include iatrogenic diseases that have occurred through medical interventions such as neurosurgery and exposure to contaminated pituitary hormone preparations. Other human acquired diseases include kuru, which was restricted to the Fore region of Papua New Guinea and was transmitted through cannibalistic mortuary feasts. Following the BSE epidemic in the UK a new human prion disease, variant CJD (vCJD), was recognised as an acquired prion disease cause by exposure to BSE-contaminated material. Different phenotypes may be explained by the existence of multiple prion strains which in experimental animals may be distinguished by their characteristic incubation times, neuropathology and biochemistry.

Susceptibility to prion diseases may be determined by many factors including genetic background. In human disease, the strongest genetic susceptibility factor occurs at a common variant within the prion protein (PrP) itself at codon 129 (M129V) [2]–[6]. In mice, aspects of prion disease susceptibility can be modelled using incubation time as a quantitative trait. As with human disease, the main genetic determinant of incubation time in mouse is variation in the prion gene, *Prnp*, where *Prnp^a^* (108-Leu, 189-Thr) and *Prnp^b^* (108-Phe, 189-Val) are associated short and long incubation times respectively [7]–[10]. Although *PRNP* plays a major role in genetic susceptibility, it has long been suspected that other genes also contribute to the observed natural variation. The first human genome wide association study (GWAS) for variant CJD (vCJD) confirmed this by implicating an additional two independent genetic loci [6]. In mice, quantitative trait locus (QTL) mapping studies have also successfully identified multiple loci across the genome that influence incubation time [11]–[15].

The GWAS study of Mead *et al* found a genome-wide significant association between vCJD and the SNP *rs6794719* which maps upstream of the gene *RARB* (retinoic acid receptor beta) on chromosome 3 (P = 1.9×10^−7^). This was replicated in a small sample of patients with iatrogenic CJD (P = 0.03) but not with sporadic CJD or kuru. A SNP upstream of *STMN2* (stathmin-like 2, the gene that encodes SCG10), *rs1460163*, on chromosome 8, was also associated with vCJD, although this did not reach the threshold for genome-wide significance (P = 5.6×10^−5^). The *rs1460163* finding was not replicated in sporadic CJD, however, an association was seen with kuru incubation time (p = 0.017) and resistance to kuru (p = 2.5×10^−4^). A standard approach for GWAS is to seek replication of these data in an independent cohort of vCJD patients, however, the rarity of the disease prohibits this and alternative methods must be sought.

We have previously used a heterogeneous stock (HS) of mice inoculated with RML mouse adapted scrapie prions to fine map and identify prion disease incubation time candidate genes [16], [17]. This resource can also be used in a genome-wide association study or to look at individual candidate genes [18]. We therefore selected *Rarb* and *Stmn2* as candidate genes to see whether we could verify the vCJD data in our mouse model.

The commoner neurodegenerative diseases, such as Alzheimer's disease (AD) also involve accumulation of misfolded proteins and it is increasingly recognised that prion-like mechanisms may be relevant in their pathogenesis [19]. Genes affecting protein homeostasis may therefore be generically involved in neurodegenerative diseases. In addition, there is evidence for a direct role of the prion protein in AD pathogenesis [20]. It is therefore reasonable to propose that susceptibility genes for AD may also be relevant for prion diseases. Two large GWAS for AD have identified *CLU*, *PICALM* and *CR1* as susceptibility genes [21], [22]. Clusterin (*Clu*) was considered to be of particular interest as it has already been implicated in prion disease in that it has been shown to bind PrP and co-localise with PrP^Sc^ in plaques [23], [24]. Further, a Clusterin knockout mouse model shows an increase in incubation time following inoculation with BSE prions [25]. We therefore also included the mouse orthologs of the AD susceptibility genes in our association study in the HS mice. To further verify our candidate genes we also looked at mRNA expression levels by real time RT-PCR in both normal and end-stage RML prion-infected mice.

## Materials and Methods

### Mice

Northport HS mice were supplied by R. Hitzemann (Portland, Oregon, USA). These were provided as 28 pairs at generation 35. The offspring were mated semi-randomly, avoiding shared grandparents, to obtain 49 mating pairs. Offspring from these pairs (generation 37) were phenotyped for prion disease incubation time (n = 1052). Inbred lines of mice were obtained from Harlan, UK (Bicester, UK) with the exception of RIIIS/J and C57BL/6J which were obtained from the Jackson Laboratory (Bar Harbor, Maine, USA).

### Prion inoculation and phenotyping

Mice were anaesthetized with isofluorane/O_2_ and inoculated intra-cerebrally into the right parietal lobe with 30µl Chandler/RML prions as previously described [11]. Mice were examined daily for clinical signs of prion disease and were culled once a definitive diagnosis had been made. Criteria for defining scrapie in mice were as previously described [26]. Incubation time was defined as the number of days from inoculation to the onset of clinical signs. All procedures were conducted in accordance with institutional, UK and international regulations and standards on animal welfare. Ethical approval was granted by the MRC Prion Unit ethics committee and carried out under UK Home Office licence PPL70/6454.

### PCR and sequencing

DNA for each of the heterogeneous stock parental lines was obtained from the Jackson Laboratory (Bar Harbor, Maine, USA). PCR and sequencing reactions were carried out as previously described [17] and run either on a MegaBACE1000 (Amersham Biosciences) or 3730 capillary sequencer (Applied Biosystems).

### Genotyping

DNA was extracted from the HS cross mice as previously described [17]. SNP genotyping was carried out using the Allelic Discrimination function on a 7500 Fast Real-time PCR machine (Applied Biosystems) using RoxMegaMix Gold (Microzone, Ltd) as previously described [17] Primers and probes are shown in Table S2 in File S1.

### Real-time RT-PCR

RNA was extracted from whole brains from either uninfected (6–8 weeks old) or RML prion-infected terminally sick mice and reverse transcribed using AMV reverse transcriptase and random primers as previously described [26]. Control reactions were carried out with no reverse-transcription for each sample to ensure no genomic DNA contamination of the RNA preparation. Real-time RT-PCR reactions were carried out on a 7500 Fast Real-time PCR System (Applied Biosystems) as previously described [26]. All gene specific probes were duplexed in turn with each of three endogenous controls (*GAPDH*, *β-actin* and *Thy-1* [17]. All reactions were carried out in triplicate. See Table S5 in File S1 for primer and probe details.

### Statistical analysis

All genotyping data were analysed using the Kruksal-Wallis non-parametric ANOVA (genotypes) and the allelic test used was the Mann-Whitney test. For quantitative RT-PCR all data passed a normality test and was therefore statistically evaluated using a two-tailed t-test.

## Results

### *Mmu14* association study

The vCJD GWAS of Mead *et al* identified a significant association with the SNP *rs6794719* on HSA 3 [6]. *RARB* was reported to be the nearest gene, although the SNP is not in linkage disequilibrium with the coding region. We therefore chose *Rarb* as a candidate gene for an association study in a mouse heterogeneous stock. As previously described, we used the Northport HS which is generated from the parental lines A/J, AKR/J, BALB/cJ, C3H/HeJ, C57BL/6J, CBA/J, DBA/2J and LP/J [16], [17], [27]. Incubation times following intra-cerebral inoculation with Chandler/RML prions were collected for n = 1052 mice at generation 37. The incubation times conformed to a normal distribution (Anderson-Darling Normality test) with a mean of 147±15 (s.d.) with a range of 103–229 days [16].

In order to identify polymorphisms for genotyping, and determine whether these may be of functional significance, we sequenced *Rarb* in the HS parental lines. Sequencing was not exhaustive but included the open reading frame, exon-intron boundaries, untranslated regions and regions predicted by PROSCAN to contain promoter elements (http://www-bimas.cit.nih.gov/molbio/proscan). Eleven polymorphisms were identified, mostly in intronic or untranslated regions (Table S1 in File S1). One polymorphism (A/C) was identified in exon 3, however, this did not result in an amino acid change (R107R). Ten of the eleven polymorphisms, including R107R, represent the same strain distribution pattern (allele A = A, AKR, BALB, C57; allele C = C3H, CBA, DBA, LP) (Table 1, Table S1 in File S1). We therefore selected the R107R SNP for genotyping, in the HS mice, as a representative of this strain distribution pattern. This was confined to the extreme 20% of both ends of the incubation time distribution (approximately n = 400) as this contains most of the power available in the cross (Table S2 in File S1) [28], [29]. Genotypic data were analysed by the Kruksal-Wallis non-parametric ANOVA and showed a highly significant association with prion disease incubation time (P = 0.0005), where the AA genotype was associated with a short incubation time (142±1.7) and the CC genotype with a longer incubation time (152±4.0). An allelic test (Mann-Whitney) also showed a highly significant association (P = 0.0002) (Table 2, Table S3 and S4 in File S1).

**Table 1:** Major strain distribution pattern for HS mice.

Genes	Strain distribution pattern	Comment
*Rarb*	(A, AKR, BALB, C57) (C3H, CBA, DBA, LP)	Exon 3, intronic and 3′UTR
*Thrb*	(A, AKR, BALB, C57) (C3H, CBA, DBA, LP)	Exon 6, intronic and 3′UTR
*Stmn2*	(A, BALB, C3H, CBA) (AKR, C57, DBA, LP)	3′UTR
*Clu*	(A, AKR, BALB, CBA) (C3H, C57, DBA, LP)	Intronic
*Picalm*	(A, AKR, BALB) (C3H, C57, CBA, DBA, LP)	Intronic
	(A, AKR, BALB, C3H, CBA, DBA, LP) (C57)	Exon 11, intronic and 3′UTR

**Table 2:** SNP genotyping in HS mice.

Gene	Polymorphism	Genotypic test p-value (n)	Allelic test p-value
*Rarb*	Exon 3 R107R A/Crs48898775	0.0005 (379)	0.0002
*Thrb*	Exon 6 V384V G/Ars16822230	0.0013 (399)	0.0005
*Stmn2*	Exon 5 3′UTR G/Ars4223708	0.0432 (396)	0.0129
*Clu*	Intron 8 C/Grs31071599	0.96 (400)	0.79
*Picalm*	Intron 9 G/Ars31101448	0.91 (368)	0.74
*Picalm*	Exon 11 I374I A/Grs32273942	0.67 (394)	0.54

All polymorphisms were analysed by allele discrimination using a 7500 Fast real time PCR system (Applied Biosystems). For probe details see Table S2 in File S1. For all genotypes, the statistical test used was the Kruksal-Wallis non-parametric ANOVA. The allelic test used was the Mann-Whitney test. dbSNP identifiers are provided with the polymorphism descriptions. Approximately 400 samples were genotyped for each SNP however, the precise number for which genotypes were collected varied due to random technical failure.

Further interrogation of the public databases showed that although *RARB* transcripts mapped within approximately 0.4Mb of *rs6794719*, another gene, *THRB* (thyroid hormone receptor beta), mapped within 0.3Mb (http://genome.ucsc.edu/ February 2009 (GRCh37/hg19) assembly). The resolution of the HS cross at generation 37 is approximately 1–2cM which generally precludes mapping to the level of an individual gene, however, analysis of strain distribution patterns may allow a more detailed view of individual associations. The gene order of the HSA3 *RARB* –*THRB* locus is also conserved in mouse (*Mmu14*) therefore we also analysed *Thrb* in our cross. Sequencing the HS parental lines was carried out as described for *Rarb*. Forty six polymorphisms were identified most of which were in non-coding regions (Table S1 in File S1). Forty two of these displayed the same strain distribution as seen for *Rarb* (allele 1 = A, AKR, BALB, C57; allele 2 = C3H, CBA, DBA, LP) (Table 1). This conservation of strain distribution pattern across both genes suggests that the *Rarb-Thrb* region of *Mmu14* is linked in this cross and will not enable us to distinguish between the contribution of individual genes. To test this hypothesis, we genotyped SNP THRBX6 V384V (Table S1 and S2 in File S1) as a representative of this strain distribution pattern. Statistical analysis showed a highly significant association between *Thrb* and prion incubation time with a genotype association of P = 0.0013 and an allelic association of P = 0.0005 (Table 2 and Table S3 and S4 in File S1). Thus, we conclude that the *Rarb-Thrb* region of *Mmu14* shows a highly significant association with prion disease incubation time in mice, however, we are unable to separate the effect of both genes due to their physical proximity.

### *Rarb* mRNA expression

**Figure 1:**
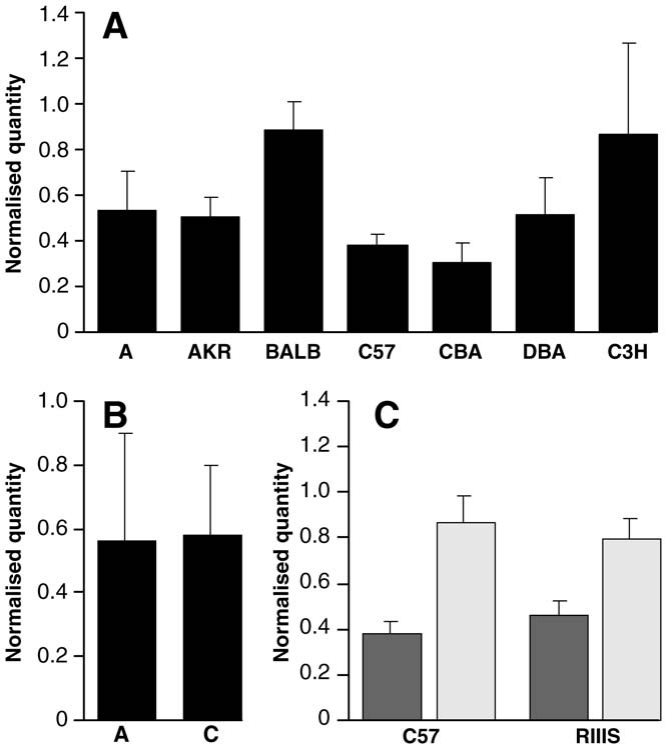
*Rarb* mRNA expression in mouse brain. Quantification of *Rarb* mRNA expression in whole brain by real-time RT-PCR. cDNA was prepared from the whole brains of uninfected 6–8 week old male mice or mice at the terminal stages of prion disease (Chandler/RML inoculated). N = 6 for all groups and samples were run in triplicate. All samples were duplexed for *Rarb* (Fam-label) and an endogenous control *GAPDH*, *β-actin* or *Thy-1* (Vic-label). Expression level is expressed in arbitrary units as normalised by the geometric mean of the quantity of the endogenous controls (*y*-axis). Error bars represent the standard error of the mean. **A**. *Rarb* mRNA expression level for parental strain of the HS mice (except LP). **B**. *Rarb* mRNA expression level grouped by allele at SNP RARBX3 R107R (A/C) (A = A, AKR, BALB, C57; C = C3H, CBA, DBA). No significant difference was observed between the groups (P = 0.87) **C**. Comparison of *Rarb* mRNA levels in uninfected (dark bars) and mice at the terminal stage of disease (light bars). Significant differences are seen between normal and terminally sick mice (P = 3.0×10^−6^ and P = 3.1×10^−5^ for C57BL/6 and RIIIS/J respectively).

No coding changes were observed in the *Rarb* ORF and no clear functions were obviously associated with the non-coding SNPs, therefore, we looked at *Rarb* mRNA expression levels in mouse brains from the parental lines of the HS (except LP) to see whether expression level could be associated with genotype. mRNA was prepared from whole brains of 6–8 week old mice and *Rarb* transcripts were quantified using real-time RT-PCR (Table S5 in File S1). Expression levels varied between the inbred lines (Figure 1A), however, when the data were grouped according to RARBX3 R107R genotype (A/C) no significant differences were seen (P = 0.87) (Figure 1B). We also compared expression levels in brains taken from animals at end stage prion disease (Chandler/RML) and uninfected controls. Samples were compared across two different inbred lines of mice (C57BL/6J and RIIIS/J) that represent both “long” and “short” incubation times respectively. For both lines, an approximately two-fold increase in expression level was observed in infected animals (P = 3.0×10^−6^, P = 3.1×10^−5^ for C57BL/6J and RIIIS/J respectively) (Figure 1C). This contrasts to the findings of Mead *et al*, that showed no difference in *Rarb* expression levels between uninfected and infected mouse neuronal (GT-1) cells in a microarray study [6]. Because no allele-specific expression differences were detected for *Rarb*, we carried out a similar study for *Thrb* to see if this could be used as a means of distinguishing between the two genes. As observed for *Rarb*, expression levels varied between inbred lines, however, when grouped by the genotype of SNP THRBX6 V384V (G/A) no significant differences were seen (P = 0.3554) (Figure S1A and S1B in File S1). Similarly, an approximately two-fold increase in expression level was seen in infected animals (P = 4.0×10^−5^, P = 7.7×10^−4^ for C57BL/6J and RIIIS/J respectively) (Figure S1C in File S1). Thus, mRNA expression analysis of *Rarb* and *Thrb* was unable to distinguish between the two candidate genes.

### *Stmn2* association study

Mead *et al* also reported an association between vCJD and SNP *rs1460163* which is located within 0.3Mb of the gene *STMN2* [6]. Although the association with *rs1460163* was replicated in kuru, the association in vCJD did not reach the threshold for genome-wide significance. We therefore sought to investigate these findings by carrying out an association study with *Stmn2* in our HS mice as described for *Rarb* and *Thrb*. Sequencing the parental lines revealed only 3 SNPs all of which were in the 3′UTR and represented the same strain distribution pattern (Table 1 and Table S1 in File S1) (allele 1 = A, BALB, C3H, CBA; allele 2 = AKR, C57, DBA, LP). SNP STMN3U1 (Table S1 in File S1) was selected as a representative SNP for genotyping in the HS mice (Table S2 in File S1). Genotype data reached a nominal level of significance (P = 0.04) where the GG genotype (allele 2) was associated with a short incubation time (144±1.6) and the AA genotype (allele 1) with a longer incubation time (146±3.3). The heterozygotes showed a longer incubation time of (148±1.9) however, this is not statistically significant. The allelic test also showed a significant association (P = 0.0129) (Table 2, and Table S3 and S4 in File S1).

### *Stmn2* mRNA expression

**Figure 2:**
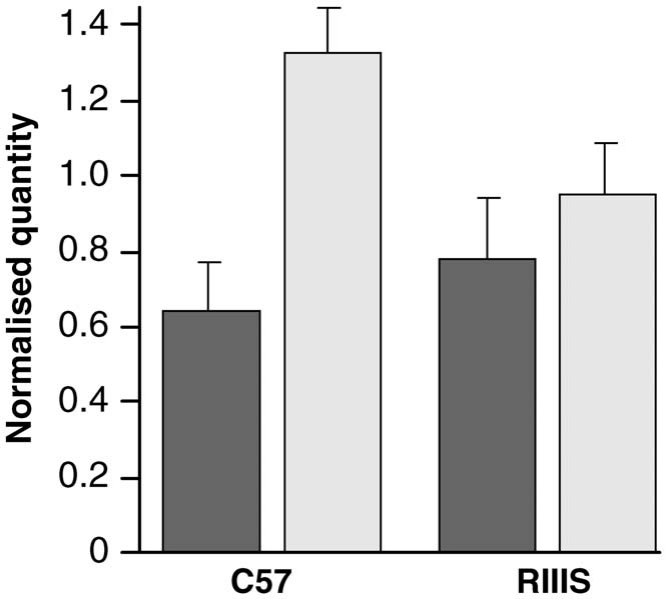
Stmn2 mRNA expression in mouse brain. Comparison of *Stmn2* brain mRNA expression between uninfected and prion-infected mice at the terminal stage of disease (Chandler/RML inoculated) by real-time RT-PCR. N = 6 for all groups and samples were run in triplicate. All samples were duplexed for *Rarb* (Fam-label) and an endogenous control *GAPDH*, *β-actin* or *Thy-1* (Vic-label). Expression level is expressed in arbitrary units as normalised by the geometric mean of the quantity of the endogenous controls (*y*-axis). Error bars represent the standard error of the mean. Dark grey and white bars represent uninfected and infected mice respectively. A significant difference is seen between normal and terminally sick mice for C57BL/6J (P = 2.0×10^−6^) but not for RIIIS/J (P = 0.08).

The human GWAS results for *STMN2* were partly corroborated by microarray data showing a 30-fold decrease in *Stmn2* expression in prion-infected mouse neuronal cells (GT-1) relative to uninfected cells [6]. Although prion-infected cell lines are a useful model they may not represent the *in vivo* situation in the mouse brain. We therefore compared *Stmn2* mRNA expression levels in uninfected and at end stage Chandler/RML prion-infected mouse brains by real time RT-PCR. (Table S5 in File S1). In contrast to the results seen in GT-1 cells, expression in C57BL/6J RML prion-infected brains was significantly increased (P = 2.0×10^−6^). However, this finding may be specific to the C57BL/6J genetic background as no significant difference was seen for RIIIS/J (P = 0.08) (Figure 2). No significant difference was observed in uninfected mouse brains between the two inbred lines (P = 0.12).

### *Clu*, *Picalm* and *Cr1* association study

To test whether AD susceptibility genes are also quantitative trait genes for prion disease incubation time in mice we analysed *Clu*, *Picalm* and *Cr1* in our HS mice as described above [21], [22]. No polymorphisms were found for *Cr1* therefore we were unable to proceed with any further analysis. One intronic SNP was identified in *Clu*, which was used for genotyping in the HS mice (Table S1 and S2 in File S1). No significant association with prion disease incubation time was seen for either genotypic (P = 0.96) or allelic data (P = 0.79) (Table 2 and Table S3 and S4 in File S1). Seventeen polymorphisms were found in *Picalm* one of which was a synonymous change in exon 11 (I374I) (Table S1 in File S1). Two major strain distribution patterns were identified (Table 1), therefore both of these were tested by genotyping in the HS mice (Table S2 in File S1). The representative SNPs tested were from intron 9 and exon 11. No significant association for prion disease incubation time was seen for either SNP by genotype or allele (Table 2 and Table S3 and S4 in File S1).

## Discussion

Human GWAS studies in neurodegenerative diseases have now identified multiple susceptibility loci. However, a major challenge is to validate these data [6], [21], [22], [30]. The first step is usually to conduct large-scale replication studies in independent cohorts, but this is not possible with a rare disease such as vCJD. We therefore sought to replicate these findings in an alternative system. Failure to replicate human susceptibility factors in mouse may however occur for several reasons, including prion strain differences and the fact that the mouse model we are using here only looks at one aspect of susceptibility, incubation time following intra-cerebral challenge. However, unlike other neurodegenerative diseases, mice are naturally susceptible to prion disease and recapitulate the neuropathology of human disease. In comparing genes identified in vCJD patients and RML inoculated mice we are aware that there may be several susceptibility genes that are not shared, however, our ultimate goal is to identify genes that influence the fundamental processes in prion disease which we would expect to be shared across all strains. Additionally, the remarkable consistency of prion incubation periods within an inbred mouse lines provides a powerful model to map relevant genes [11]. *Hectd2* is an example of a susceptibility gene first identified in mouse that is also associated with the human diseases vCJD and kuru [16].

Although Mead *et al* highlighted *RARB* as being the closest gene to *rs6794719*, *THRB* also maps within 0.5Mb. We have shown a significant association of the *Rarb-Thrb* locus with prion disease incubation time thereby validating the human GWAS data. Due to the resolution available in the HS mice we were unable to distinguish between the effect of the two candidate genes. Based on previous reports, *Rarb* appears to be the most promising candidate. *Thrb* has not previously been associated with prion disease although thyroid stimulating hormone has been implicated in PrP mRNA expression [31]. Retinoic acid, the Rarb ligand, has been shown to regulate the *Prnp* promoter, influence PrP^c^ (cellular PrP) levels and cause an increase in PrP^res^ (proteinase K resistant abnormal isoform of PrP) accumulation in ScN2a cells [32], [33].

No Rarb coding variants were detected by sequencing. One polymorphism was a synonymous change in the ORF, R107R. Other polymorphisms were intronic or in untranslated regions with no ascribed function. Our sequencing was not exhaustive therefore it is possible that these SNPs do not represent the functional variants but are acting as markers for functional polymorphisms that were not identified by our sequencing. While our expression study did not detect any significant difference associated with genotype, subtle differences may be masked by the effect of other regulators, possibly on other chromosomes and by the effects of multiple cell types present in whole brain samples. Some significant differences were seen between strains suggesting that other *cis*-acting loci are involved in the overall gene expression level.

Our analysis does not rule out the possibility of allele-specific effects on splicing and other regulatory events.

Although *STMN2* did not reach the threshold for genome-wide significance in the human vCJD GWAS study, supporting evidence was provided from replication in the acquired prion disease from Papua New Guinea, kuru [6]. Similarly, in the HS mice, *Stmn2* gave a significant association with prion disease incubation time (P = 0.04). A level of significance across a range of studies representing different hosts and prions strains suggests that *Stmn2* may well be worthy of further investigation.

In mouse brain, we have shown that *Rarb*, *Thrb* and *Stmn2* are significantly increased in prion-infected as compared to uninfected animals although for *Stmn2* this effect was not replicated in RIIIS/J mice. This is in contrast to the data reported for prion infected GT-1 cells where no difference was seen for *Rarb* and a 30-fold decrease was shown for *Stmn2* [6]. It is likely that this reflects the difference between prion propagation *in vitro* in an immortalised single cell line and the situation *in vivo* at end-stage disease where cells of different types are present, neurons are terminally differentiated and undergoing substantial stress leading to death. The use of end-stage brains where significant pathology is present may mask the level of up or down regulation. In this study we compared terminally sick mice to 6–8 week old mice therefore we cannot exclude the possibility that some of the observed changes may also be age related. In addition, there may be differences due to genetic background as GT-1 cells are derived from transgene induced hypothalamic neuronal tumours in mice generated from F2 C57BL/6J×BALB/cJ embryos [34].

The increasingly apparent mechanistic overlap between neurodegenerative diseases suggested that susceptibility genes found in AD GWAS may also be relevant to prion disease. Although *Picalm* and *Cr1* have not previously been implicated in prion disease there is ample evidence to suggest that clusterin may be involved [23], [24]. Data showing that a clusterin knockout mouse inoculated with BSE prions has a significantly increased incubation time suggested that *Clu* was a particularly good candidate quantitative trait gene for prion disease incubation time [25]. Although previous data clearly implicates clusterin in prion disease, our data show that it does not contribute to the natural variation observed in the RML model of mouse incubation time. It is possible that the effect of clusterin on incubation may be prion strain specific and an association may be seen with other strains particularly BSE. Our data provides significant further evidence to support a role for the *Rarb* genetic locus in prion disease and provides some supporting evidence that S*tmn2* may also be implicated. Functional assessment of both genes will be required to confirm these genetic data.

## Supporting Information

File S1 (DOC)
