# Supplementary material for: The Retinoic Acid Receptor Beta (Rarb) Region of Mmu14 Is Associated with Prion Disease Incubation Time in Mouse
Source: PLoS One. 2010 Dec 6;5(12):e15019. doi: 10.1371/journal.pone.0015019 (PMC2997791; doi:10.1371/journal.pone.0015019)
Supplement: File S1 — (DOC) [file pone.0015019.s001.doc]

**Supplementary information**

**Figure S1**

**
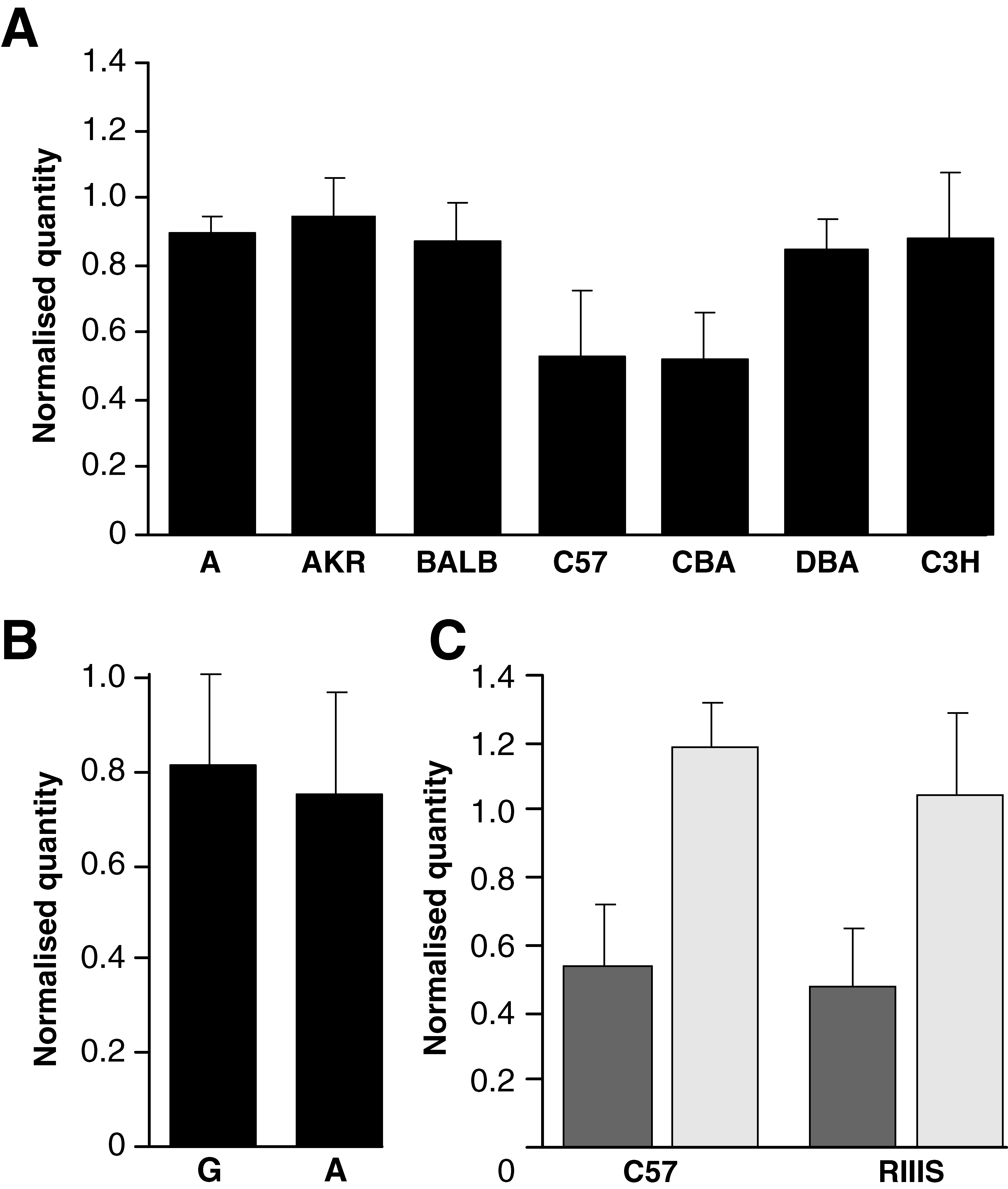
**

**Figure S1**

***Thrb* mRNA expression**

Quantification of *Thrb* mRNA expression in whole brain by real-time RT-PCR. cDNA was prepared from the whole brains of uninfected 6-8 week old male mice or mice at the terminal stages of prion disease (Chandler/RML inoculated). N=6 for all groups and samples were run in triplicate. All samples were duplexed for *Thrb* (Fam-label) and an endogenous control *GAPDH, β-actin* or *Thy-1* (Vic-label). Expression level is expressed in arbitrary units as normalised by the geometric mean of the quantity of the endogenous controls (*y*-axis). Error bars represent the standard error of the mean. **A**. *Thrb* mRNA expression level for parental strain of the HS mice (except LP). **B.** *Thrb* mRNA expression level grouped by allele at SNP THRBX6 V384V (G/A) (G=A, AKR, BALB, C57; A=C3H, CBA, DBA). No significant difference was observed between the groups (P=0.36) **C.** Comparison of *Thrb* mRNA levels in uninfected (dark bars) and mice at the terminal stage of disease (light bars). Significant differences are seen between normal and terminally sick mice (P=4.0x10^-5^ and P=7.7x10^-4^ for C57BL/6 and RIIIS/J respectively, t-test). No statistically significant differences are seen between either uninfected or infected C57BL/6 and RIIIIS/J.

**Table S1**

Summary of SNPs from candidate genes

| **SNP name** | **A** | **AKR** | **BALB** | **C3H** | **C57** | **CBA** | **DBA** | **LP** |
| --- | --- | --- | --- | --- | --- | --- | --- | --- |
| ***Rarb*** |  |  |  |  |  |  |  |  |
| RARBU1A | A | A | A | C | A | C | C | C |
| RARBU1B | Ins | Ins | Ins | del | Ins | del | del | del |
| RARBIN1A | G | G | T | T | T | T | T | T |
| RARBIN1B | A | A | A | T | A | T | T | T |
| RARBIN1C | A | A | A | G | A | G | G | G |
| RARBX3 R107R | A | A | A | C | A | C | C | C |
| RARBIN3A | C | C | C | T | C | T | T | T |
| RARBIN3B | C | C | C | T | C | T | T | T |
| RARBIN3C | C | C | C | G | C | G | G | G |
| RARBIN3D | T | T | T | G | T | G | G | G |
| RARB3U | T | T | T | C | T | C | C | C |
| ***Thrb*** |  |  |  |  |  |  |  |  |
| THRBU1A | C | C | C | T | C | T | T | T |
| THRBU1B | G | G | A | G | G | G | G | G |
| THRBIN1A | A | A | A | G | A | G | G | G |
| THRBIN1B | Ins | Ins | Ins | del | Ins | del | del | del |
| THRBIN1C | A | A | A | G | A | G | G | G |
| THRBIN1D | 5 | 5 | 5 | 7 | 5 | 7 | 7 | 7 |
| THRBIN1E | C | C | C | G | C | G | G | G |
| THRBIN3A | A | A | A | G | A | G | G | G |
| THRBIN3B | T | T | T | C | T | C | C | C |
| THRBIN3C | C | C | C | T | C | T | T | T |
| THRBIN3D | T | T | T | A | T | A | A | A |
| THRBIN3E | T | T | T | C | T | C | C | C |
| THRBIN3F | T | T | T | C | T | C | C | C |
| THRBIN3G | G | G | G | T | G | T | T | T |
| THRBIN3H | A | A | A | G | A | G | G | G |
| THRBIN4A | T | T | T | C | T | C | C | C |
| THRBX6 V384V | G | G | G | A | G | A | A | A |
| THRBIN6A | C | C | C | T | C | T | T | T |
| THRB3UA | C | C | C | T | C | T | T | T |
| THRB3UB | G | G | G | A | G | A | A | A |
| THRB3UC | C | C | C | A | C | A | A | A |
| THRB3UD | G | G | G | A | G | A | A | A |
| THRB3UE | T | T | T | C | T | C | C | C |
| THRB3UF | C | C | C | T | C | T | T | T |
| THRB3UG | G | G | G | A | G | A | A | A |
| THRB3UH | T | T | T | C | T | C | C | C |
| THRB3UI | A | A | G | G | A | G | G | G |
| THRB3UJ | G | G | G | A | G | A | A | A |
| THRB3UK | A | A | A | T | A | T | T | T |
| THRB3UL | T | T | T | C | T | C | C | C |
| THRB3UM | C | C | C | T | C | T | T | T |
| THRB3UN | G | G | G | A | G | A | A | A |
| THRB3UO | T | T | T | C | T | C | C | C |
| THRB3UP | T | T | T | G | T | G | G | G |
| THRB3UQ | T | T | A | A | T | A | A | A |
| THRB3UR | T | T | T | C | T | C | C | C |
| THRB3US | C | C | C | T | C | T | T | T |
| THRB3UT | C | C | C | G | C | G | G | G |
| THRB3UU | T | T | T | C | T | C | C | C |
| THRB3UV | del | del | del | T | del | T | T | T |
| THRB3UW | G | G | G | A | G | A | A | A |
| THRB3UX | G | G | G | C | G | C | C | C |
| THRB3UY | A | A | A | G | A | G | G | G |
| THRB3UZ | G | G | G | A | G | A | A | A |
| THRB3UA2 | T | T | C | C | T | C | C | C |
| THRB3UB2 | T | T | T | C | T | C | C | C |
| ***Stmn2*** |  |  |  |  |  |  |  |  |
| STMN3U1 | A | G | A | A | G | A | G | G |
| STMN3U2 | G | A | G | G | A | G | A | A |
| STMN3U3 | 5 | 4 | 5 | 5 | 4 | 5 | 4 | 4 |
| ***Clu*** |  |  |  |  |  |  |  |  |
| CLUIN8 | G | G | G | C | C | G | C | C |
| ***Picalm*** |  |  |  |  |  |  |  |  |
| PICALMIN1 | C | C | C | T | T | T | T | T |
| PICALMIN2 | C | C | C | C | T | C | C | C |
| PICALMIN3 | C | C | C | C | T | C | C | C |
| PICALMIN5 | del | del | del | del | T | del | del | del |
| PICALMIN6 | del | del | del | del | ATT | del | del | del |
| PICALMIN7 | G | G | G | G | A | G | G | G |
| PICALMIN8 | G | G | G | G | A | G | G | G |
| PICALMIN9 | A | A | A | G | G | G | G | G |
| PICALMX11  I374I | G | G | G | G | A | G | G | G |
| PICALMIN13A | GTTT | GTTT | GTTT | del | del | del | del | del |
| PICALMIN13B | A | A | A | A | G | A | A | A |
| PICALMIN14 | A | A | A | G | G | G | G | G |
| PICALMIN17 | del | del | del | TAG | TAG | TAG | TAG | TAG |
| PICALM3UA | A | A | A | G | G | G | G | G |
| PICALM3UB | A | A | A | T | A | T | T | T |
| PICALM3UC | A | A | A | G | G | G | G | G |
| PICALM3UD | A | A | A | A | C | A | A | A |
|  |  |  |  |  |  |  |  |  |
|  |  |  |  |  |  |  |  |  |
|  |  |  |  |  |  |  |  |  |
|  |  |  |  |  |  |  |  |  |

Abbreviations: IN=intron, X=exon, U = upstream of exon 1, 3U=3’UTR, del=deletion, Ins=insert. SNPs highlighted in red were used for further genotyping in the HS mice. No SNPs were observed in gene *Cr1*. For STMN3U3 4 and 5 refer to the number of A bases. For THRB1N1D 5 and 7 refer to the numbers of T bases.

**Table S2**

**Primer and probe sequences for SNP genotyping** by allelic discrimination

| **Gene** | **Polymorphism** | **Primers and probes** |
| --- | --- | --- |
| *Rarb* | Exon 3 R107R A/C | GGTCTCTCATGAAGCTAGCTATTTATCTG  CAGTTCTTATCTCGATGGCAAGTG  Fam-TTTTTCCGC**A**GAAGTAT  Vic – TTTTTCCGC**C**GAAGTA |
| *Thrb* | Exon 6 V384V G/A | CTGTCATCTTTCAACCTGGATGAC  CCTTGAGAATCTCAGCACACTCAC  Fam-TGAAGT**C**GCCCTGC  Vic-CTGAAGT**T**GCCCTGC |
| *Stmn2* | Ex5 3’UTR G/A | GCGCTACACATGTGTTTGGGT  GCTTCTACCCTGACTGAGCTCAGA  Fam – CATGCAC**T**TGTTTCT  Vic - ACATGCAC**C**TGTTTC |
| *Clu* | Intron 8 C/G | CCTTTCTGTGACCCCAGGG  GCTCTGGCCTCAGGGATAGG  Fam-TGGTAAACAAGA**C**CCTG  Vic-CTGGTAAACAAGA**G**CCT |
| *Picalm* | Intron 9 G/A | ggttatctgttagataggtttaagtaatatgagtg  aattaaccccatggaatcctgtt  Fam-actgtcactagtaatta**g**ttaca  Vic-actgtcactagtaatta**a**ttacatta |
| *Picalm* | Exon 11 I374I A/G | cctcagcagggggaataatga  atccttattgcctgttgtacctgtt  Fam-accagccat**t**gacata    Vic-accagccat**c**gacata |

All SNPs were genotyped using a 7500 Real Time PCR machine (Applied Biosystems). All probes were MGB labelled probes from Applied Biosystems. Bases in bold represent the SNP.

**Table S3**

Details for genotype statistical analysis

| **Gene** | **Polymorphism** | **Genotype incubation times**  **Days + SEM (n)** | **HS p-value (ANOVA)** |
| --- | --- | --- | --- |
| *Rarb* | Exon 3 R107R A/C | AA 142 + 1.7 (182)  AC 148 + 1.8 (166)  CC 152 + 4.0 (31) | P=0.0005 (n=379) |
| *Thrb* | Exon 6 V384V G/A | GG 142 + 1.7 (186)  GA 148 + 1.7 (179)  AA 150 + 3.8 (34) | P=0.0013 (n=399) |
| *Stmn2* | Exon 5 3’UTR G/A | GG 144 + 1.6 (192)  GA 148 + 1.9 (166)  AA 146 + 3.3 (38) | P=0.0432 (n=396) |
| *Clu* | Intron 8 C/G | CC 145 + 2.4 (51)  CG 146 + 1.7 (185)  GG 146 + 1.9 (164) | P=0.96 (n=400) |
| *Picalm* | Intron 9 G/A | AA 145 + 2.1 (116)  AG 147 + 1.9 (162)  GG 147 + 2.2 (90) | P=0.91 (n=368) |
| *Picalm* | Exon 11 I374I A/G | CC 144 + 1.7 (188)  CT 146 + 1.8 (183)  TT 145 + 3.8 (23) | P=0.67 (n=394) |

In all cases the statistical test used was the Kruksal-Wallis non-parametric ANOVA.

**Table S4**

Details for allelic statistical analysis

| **Gene** | **Polymorphism** | **Allele incubation times**  **Days + SE** | **Allelic Test p-value** |
| --- | --- | --- | --- |
| *Rarb* | Exon 3 R107R A/C | A 144 + 1.0  C 149 + 1.5 | P=0.0002 |
| *Thrb* | Exon 6 V384V G/A | G 144 + 1.0  A 149 + 1.5 | P=0.0005 |
| *Stmn2* | Exon 5 3’UTR G/A | G 145 + 1.0  A 148 + 1.5 | P=0.0129 |
| *Clu* | Intron 8 C/G | C 146 + 1.3  G 146 + 1.0 | P=0.79 |
| *Picalm* | Intron 9 G/A | A 146 + 1.8  G 147 + 1.2 | P=0.74 |
| *Picalm* | Exon 11 I374I A/G | C 145 + 1.0  T 146 + 1.5 | P=0.54 |

In all cases the statistical test used was a Mann-Whitney test.

**Table S5**

**Primer and probe sequences for** Taqman real-time PCR expression analysis

|  |  |  |
| --- | --- | --- |
|  |  |  |
|  | | **Gene** | **Primer and Probes** | | --- | --- | | *Rarb* | Catcgagataagaactgcgtcatt  catgcccacttcaaagcactt  Fam-Cactaggaaccgatgccagtactgccg-Tamra | | *Thrb* | GCCTTAAAACCGAGACCACAGT  TTCCGTGATAAAAGCCATGCT  Fam–AAGCTGTAGAGCCCAGGCCGCC-Tamra | | *Stmn2* | GGAGCTGTCTATGCTGTCACTGA  CGTCGTAGGTGTAGATGTTGATGTT  Fam-TCCTGCTTCTACCCGGAGCCGC-Tamra | |  |
